# On Relationships between Plasma Chemistry and Surface Reaction Kinetics Providing the Etching of Silicon in CF_4_, CHF_3_, and C_4_F_8_ Gases Mixed with Oxygen

**DOI:** 10.3390/ma16145043

**Published:** 2023-07-17

**Authors:** Seung Yong Baek, Alexander Efremov, Alexander Bobylev, Gilyoung Choi, Kwang-Ho Kwon

**Affiliations:** 1 Department of Computer Science and Technology, Korea University, Sejong 30019, Republic of Korea; 100baekga@korea.ac.kr; 2 Department of Electronic Devices & Materials Technology, State University of Chemistry & Technology, 7 Sheremetevsky av., Ivanovo 153000, Russia; amefremov@yandex.ru (A.E.); prototyp16@mail.ru (A.B.); 3 Department of Control and Instrumentation Engineering, Korea University, Sejong 30019, Republic of Korea; confidencism@korea.ac.kr

**Keywords:** fluorocarbon gases, active species, ionization, dissociation, etching

## Abstract

In this work, we discuss the effects of component ratios on plasma characteristics, chemistry of active species, and silicon etching kinetics in CF_4_ + O_2_, CHF_3_ + O_2_, and C_4_F_8_ + O_2_ gas mixtures. It was shown that the addition of O_2_ changes electrons- and ions-related plasma parameters rapidly suppresses densities of CF_x_ radicals and influences F atoms kinetics through their formation rate and/or loss frequency. The dominant Si etching mechanism in all three cases is the chemical interaction with F atoms featured by the nonconstant reaction probability. The latter reflects both the remaining amount of fluorocarbon polymer and oxidation of silicon surface.

## 1. Introduction

Gaseous fluorocarbons are frequently used in plasma-forming environments for the “dry” patterning of silicon and silicon-based materials during the production of micro- and nanoelectronic devices [1,2,3]. The most comprehensive tool here is the reactive-ion etching (RIE) process that combines the chemical etching (the formation of volatile reaction products due to the interaction of etchant species with surface atoms) and the physical sputtering of the treated surface. Accordingly, usual RIE conditions suggest low gas pressures (p < 20 mTorr), high input power densities (w~0.1 W/cm^3^) in order to produce high ionization degrees for gas species as well as high ion bombardment energies ($\varepsilon_{i}$ > 100 eV) in order to overcome typical sputtering thresholds [4,5].

The specific feature of all fluorocarbon gas plasmas is the surface polymerization effect provided by nonsaturated CF_x_ radicals. This causes the formation of continuous fluorocarbon polymer film on the etched surface (as well as on any surface contacted with plasma) while the film thickness influences etching kinetics and output RIE characteristics: process rate and selectivity regarding both mask and substrate materials as well as profile shape [6,7,8]. From many published works, it can be understood that (a) the polymerizing ability for any fluorocarbon gas plasma strongly depends on the z/x ratio in the original C_x_H_y_F_z_ molecule and (b) various fluorocarbon gases being exposed to one and the same processing conditions produce etching environments with different properties in respect to RIE of Si and SiO_2_. For example, the CF_4_ (z/x = 4) plasma provides fast etching process (over 300 nm/min for Si [5,6]) with decent surface clearness but suffers from the strongly isotropic etching of silicon and low etching selectivity in the SiO_2_/Si couple [1,6,7]. Such a situation is because the combination of high density of F atoms with low density of CF_x_ (x = 1, 2) radicals in a gas phase takes place. As a result, the low polymerizing ability produces the thinness of even the noncontinuous (islandlike) polymer film on the etched surface. Oppositely, the plasma excited in C_4_F_8_ (z/x = 2) exhibits the much higher polymerizing ability because of the domination of CF_x_ radicals over F atoms in a gas phase [9,10,11]. That is why the corresponding RIE process is featured by the deposition of thick (up to tens of microns [6,7,8]) and continuous polymer film. The latter leads to relatively low etching rates with high etching restudies as well as allows one to obtain nearly vertical etching profiles (as the polymer prevents the interaction of F atoms with side walls) [4,6,7] and high SiO_2_/Si etching selectivity (as the thicker film on the oxygen-free surface reduces the etching rate for silicon through the worse access of F atoms) [6,8].

The effective method to adjust the etching/polymerization balance in given RIE process is to combine highly or moderately polymerizing fluorocarbon gas with the additive component that suppresses the polymerization. From [11,12,13,14], it can be understood that the addition of O_2_ always lowers the density of CF_x_ radicals in a gas phase through the CF_x_ + O/O(^1^D) → CF_x−1_O + F reaction family as well as causes the oxidative destruction of deposited polymer film. Though mixtures of fluorocarbons with oxygen and other additive gases have been intensively studied during the last decade, the main attention was attracted to CF_4−_ based plasmas. As a result, existing experimental and theoretical works for CF_4_ + O_2_ plasma [12,14,15,16,17] (a) provided detailed data on the influence of processing conditions on both gas-phase plasma characteristics and etching kinetics for a wide number of materials, (b) suggested adequate kinetic schemes for both gas-phase and heterogeneous reaction pathways in the presence of oxygen, and (c) confirmed the acceptable correlation between densities of plasma species obtained through experimental and modeling procedures. At the same time, other fluorocarbon gases received much less attention in respect to plasma chemistry and thus look much worse understood compared with CF_4_. In particular, CHF_3_ and C_4_F_8_ were mainly studied either in the oxygenless gas mixtures [9,18,19,20,21] or as components of CHF_3_ + Ar + O_2_ and C_4_F_8_ + Ar + O_2_ plasmas with variable Ar/O_2_ mixing ratio [10,13,14,22]. In the last case, gas mixtures always contained 50% of CHF_3_ or C_4_F_8_, so that chemical reactions with a participation of oxygen atoms occurred under an excess of the fluorocarbon component. Accordingly, such experimental conditions and related discussions situation do not provide the complete understanding of all possible oxygen-related effects taking placed in O_2_-rich plasmas. In addition, our previous works [9,10,23] have demonstrated that (a) O_2_/Ar and CF_4_/O_2_ ratios in the CF_4_ + O_2_ + Ar gas mixture produce quite different changes in plasma parameters, gas-phase composition, and etching process kinetics; and (b) steady-state densities of atomic fluorine in nonoxygenated CF_4−_, CHF_3−_, and C_4_F_8−_ based plasmas are controlled by different chemical processes. Therefore, any direct analogies between the well-studied CF_4_ + O_2_ plasma and the purely studied CHF_3_ + O_2_ or C_4_F_8_ + O_2_ plasma do not reflect adequately the situation in the last one and thus are not useful for the corresponding RIE process optimization.

The main idea of this work was to compare the properties of CF_4_ + O_2_, CHF_3_ + O_2_, and C_4_F_8_ + O_2_ plasmas under identical operating conditions as well as in a wide range of fluorocarbon/oxygen mixing ratios. Accordingly, main research efforts were focused on such questions as (1) to compare the influence of fluorocarbon/oxygen mixing ratios on electrons- and ions-related plasma parameters, (2) to figure out peculiarities of fluorine atom kinetics in both O_2_-excess and O_2_-deficient reaction regimes, and (3) to analyze how differences in plasma parameters and compositions are reflected on the RIE process for silicon. Though the silicon itself is not the typical material etched by these plasmas in real device technologies, it represents the suitable test object for investigations of etching chemistry, namely, for the comparison of various gas systems. The reasons are the availability of quite accurate data on sputter yield that allows one to separate contribution of physical and chemical etching pathways as well as the well-known chemical etching mechanism that mainly appears in a form of the spontaneous chemical reaction with F atoms [4]. Therefore, we believe that the analysis of Si etching kinetics at the nearly constant surface temperature provides the easier understanding of the heterogeneous effect related to both etching/polymerization balance and other side factors influencing the interaction of F atoms with the target surface.

## 2. Experimental and Modeling Details

### 2.1. Experimental Setup and Methods

Like in our previous works [9,10,11], plasma diagnostics and etching experiments were conducted in the planar inductively coupled plasma (ICP) reactor with cylindrical (r = 13 cm, l = 16 cm) chamber made from the anodized aluminum. Schematic diagram of experimental equipment may be found in [10]. Plasma was produced using the 13.56 MHz generator, which powered the 3.5-turn copper coil at chamber top side. Another 13.56 MHz power supply was matched with the bottom electrode to generate the negative bias voltage, $-U_{dc}$. The latter was controlled using the high-voltage probe (AMN-CTR, Youngsin Eng.) Constant input parameters were total gas flow rate (q = 40 sccm), gas pressure (p = 6 mtor), input power (W = 700 W, or ~0.8 W/cm^3^), and bias power ($W_{dc}$ = 200 W). The single variable input was represented by component fractions $y_{i}$ in CF_4_ + O_2_, CHF_3_ + O_2_, and C_4_F_8_ + O_2_ gas mixtures. These were controlled through partial gas flow rates $q_{i}$ so that an increase in $q_{O_{2}}$ from 0–30 sccm corresponded to $y_{O_{2}}$ = 0–75%. The latter surely covers both oxygen-deficient and oxygen-excess reaction regimes.

Electrons- and ions-related plasma characteristics were measured using the rf-compensated double Langmuir probe tool (DLP2000, Plasmart Inc.). The probe head was installed through the viewport on the chamber wall as well as situated in the center of reactor chamber at ~5 cm above the bottom electrode. Measured current-voltage (I–V) curves were treated according to basic statements of Langmuir probe theory for low-pressure and low-electronegative plasmas [4,24]. After this, we obtained the current density ($J_{+}$) and the electron temperature ($T_{e}$). To reduce the overall experimental error caused by the polymerization on probe tips, these were conditioned for ~5 min in 50% Ar + 50% O_2_ plasma before and after measuring each new point. Previously, we have confirmed the efficiency of this procedure to obtain adequate plasma diagnostics data in high polymerizing fluorocarbon-based plasmas [10,11,13,14]. Unfortunately, we could not provide the direct measurement of the electron energy distribution function (EEDF) because the noisy signal produced high uncertainty in its shape.

Steady-state densities of fluorine atoms were determined by the optical emission spectroscopy (OES) (AvaSpec-3648, JinYoung Tech) using the actinometry method. The emission was taken through the sidewall viewport with the quarts window, and the axial position corresponded to that for the Langmuir probe tool. For the actinometry purpose, we introduced 2 sccm (~4.5%) of Ar in each gas mixture and then, measured emission intensities (I) for well-known analytical lines, such as F 703.8 nm ($\varepsilon_{th}$ = 14.75 eV) and Ar 750.4 nm ($\varepsilon_{th}$ = 13.48 eV). From [25], it can be understood that both lines are featured by direct electron impact excitations with known process cross-sections [25] as well as exhibit very low lifetimes for corresponding excited states. The latter allows one to neglect their collisional relaxation pathway while the standard actinometrical approach [25,26] yields.

(1) $$n_{F}=n_{Ar}C_{act}\frac{I_{F}}{I_{Ar}}$$

where $n_{F}$ is the fluorine atom density, $n_{Ar}=y_{Ar}N$, $N=p/k_{B}T_{gas}$ is the total gas density at the given gas temperature $T_{gas}$, and $C_{act}$ is the actinometric coefficient. Data of Ref. [25] indicate also that (a) actinometric coefficient $C_{act}=f\left(T_{e}\right)$ keeps the almost constant value of ~2.1 at electron temperatures 3–6 eV; and (b) densities of F atoms determined from Equation (1) with the F 703.8 nm/Ar 750.4 nm intensity ratio are in good agreement with those measured by the mass spectrometry. From plasma diagnostics by Langmuir probes, it was found also that the given amount of Ar has no noticeable influence on both electron temperature and plasma density. Therefore, the presence of actinometry gas does not disturb kinetics of plasma active species while the corresponding $n_{F}$ values obtained from Equation (1) may surely be equalized with those in Ar-free plasmas.

Etching kinetics for silicon was investigated using pieces of Si (111) wafers with an average size of ~2 × 2 cm which were placed in the center of the bottom electrode. The water-flow cooling system stabilized its temperature at ~17 °C during processing times up to 5 min. To determine etching rates, a part of sample surface was masked by the photoresist (AZ1512, positive) with a thickness of ~1.5 μm. Accordingly, after each etching experiments we measured the step Δh between nonmasked and masked areas using the surface profiler Alpha-Step 500 (Tencor). 

To determine basic features of our etching system and processing conditions regarding the Si etching kinetics, we conducted a series of preliminary experiments, including plasma diagnostics by Langmuir probes and etching rate measurements. Corresponding results may briefly be summarized as follows:

1. Processing times τ up to 5 min surely provide nearly linear kinetic curves Δh=f(τ), like those obtained in [27] in given reactor and close range of processing conditions. This points out on the steady-state etching regime as well as allowed one to obtain etching rates simply as R=Δh/τ.
2. There are no differences in etching kinetics for Si samples situated at different radial positions, except weakly decreasing etching rates toward camber walls. Therefore, one can speak about the spatially independent etching mechanism while the last effect is due to nonuniform radial profiles for densities and fluxes of plasma active species [4]. The latter is kind of fundamental phenomenon for any plasma etching reactor caused by both faster generation of active species in the axial region and their effective losses on chamber walls [2,4].
3. There are no noticeable changes in Si etching rate with increasing amount of simultaneously loaded samples. The absence of loading effect means an excess of active species participating in the chemical etching pathway [4], so that the dependence of etching rate on processing conditions reflects the real heterogeneous process kinetics.
4. There are no differences in Langmuir probe diagnostics data obtained without and with sample loading. Therefore, one can neglect the influence of etching products on gas-phase plasma characteristics as well as to assume plasma to be the undisturbed source of active species.

Obviously, the last two features are due to the low sample size that limits both consumption of active species for chemical reaction and the flux of etching products leaving the surface. It important to note also that all above findings confirm those obtained in our previous works [9,10,11,13,14,20,27]. The latter assumes the applicability of earlier used statements and approaches for the analysis of experimental and model-provided data.

### 2.2. Approaches for the Analysis of Plasma Chemistry

In previous works, there were several formally successful attempts to develop comprehensive self-consistent models for oxygen-free CF_4−_, CHF_3−_, and C_4_F_8−_ based plasmas [18,19,21,28,29,30]. Corresponding computational algorithms matched the extended (including reaction sets for positive and negative ions) plasma chemistry module with the input power balance equation that finally allowed one to obtain model-predicted electron temperature and electron density. Accordingly, the comparison of these data with experimental ones provided the model adequacy criteria as well as characterized the predictive potential of the model. At the same time, one can conclude that there is not a kind of “perfect case” while the agreement between model and experiment may be worse or better for various parameters within one model and/or for different models related to one and the same gas system. In our opinion, the problem is the uncertainty in the power balance module because many principal cross-sections determining electron energy losses in collisions with non-saturated C_x_H_y_F_z_ species (for instance, vibrational and/or low-threshold electronic excitations) are not known. As such, these were either ignored or roughly evaluated using various physical approaches and analogies with “similar” species. Probably, some of those appeared to be far from the reality, so that the model performance is closely linked with fractions of corresponding components in a gas phase. In oxygen-containing plasmas, the additional problem is the lack of cross-section data for essential gas-phase products, such as FO, CFO, and CF_2_O. Such a situation does not allow to perform an adequate quantitative description for both electron energy loss channels and ionization/recombination balance as well as produces critical uncertainties in the self-consistent modeling algorithm. That is why we applied a simplified 0-dimensional (global) model that involved experimental data on $T_{e}$ and $J_{+}$ as input parameters, accounted for the neutral chemistry only, and required the limited amount of ionization rate coefficients for dominant neutral components to evaluate the effective ion mass [11,13,14]. Though it may look like a kind of downgrade compared with previous works, such an approach equalizes the model accuracy for all three gas systems and thus provides the adequate comparison of their basis properties. 

The set of chemical reaction with corresponding rate coefficients for each gas mixture was taken from our previous model-based works dealt with CF_4_ + Ar/O_2_, refs. [13,17,23] CHF_3_ + Ar/O_2_ [31] and C_4_F_8_ + Ar/O_2_ [13,32] plasmas. Earlier, these kinetic schemes have been used by several authors and demonstrated the decent similarity between model-predicted and measured F atom densities [11]. Table 1, Table 2 and Table 3 represent reduced reaction sets that include only the most important processes determining steady-state densities of neutral species. All these are either involved in the discussion or needed to trace basic reaction mechanisms. Some reactions are written in the generalized form to group those featured by similar source species and/or byproducts. As given gas systems have many common particles, Table 2 and Table 3 contain only additive reactions to be merged with those from Table 1.

Basic model approaches were formulated as follows:

1. The electron energy distribution function may be approximated by Maxwellian one. The applicability of such an assumption for the given set of plasma excitation conditions is surely supported by direct measurements of EEDF in CF_4−_ based plasmas [12,28] as well as indirectly follows from the quite acceptable agreement between model-predicted and measured plasma parameters in CHF_3−_ [21,29] and C_4_F_8−_ [18,19] based plasmas. A similar conclusion can also be made for O_2_ plasma in the absence of fluorocarbon components [33,34]. The common reason is the high ionization degree for neutral species ($n_{+}/N$ > 10^−4^, where $n_{+}$ is the total density of positive ions) that causes the essential role equilibrium electron–electron collisions in the overall electron energy balance. Accordingly, rate coefficients for electron-impact reactions (R1–R9 in Table 1, R25–R29 in Table 2 and R40–R44 in Table 3) may be obtained using fitting expressions $k=f\left(T_{e}\right)$ [12,18,28,29,33].
2. The gas temperature is mainly controlled by gas pressure (since it determines gas density, collision frequency and heat transfer coefficient) and input power (since it represents a gas heating source) [4,35,36] as well as exhibits rather close values for many molecular gases [34]. In experiments, we found that the temperature of external chamber wall is almost not sensitive to variations in both gas mixing ratios and the type of the fluorocarbon components for any fixed “plasma on” time. The latter confirms that p, W = const really provides $T_{gas}$ ≈ const. Similarly to our previous works [11,14], we took the value of 600 K (as it was measured for both CF_4_ and O_2_ plasmas for given gas pressure and input power density of ~0.7 W/cm^3^ [35]) and then obtained rate coefficients for gas-phase atom–molecular reactions (R10–R21 in Table 1, R30–R36 in Table 2 and R45–R47 in Table 3) using Arrhenius-like expressions from [36].
3. The decay of atoms and radicals on chamber walls (R22–R24 in Table 1, R37–R39 in Table 2, and R48 in Table 3) follows the Eley–Rideal (the first-order in respect to gas-phase species) recombination kinetics. In this case, corresponding rate coefficients are $k\approx \left(r+l\right)\upsilon_{T}\gamma /2rl$, where r and l are radial and axial dimensions of the reactor chamber, respectively, $\upsilon_{T}=\sqrt{8k_{B}T_{gas}/\pi m}$ is the thermal velocity for the particle with a mass of m, and γ is the recombination probability [12,18,28,29,30]. For simplicity, we assumed all recombination probabilities to be not sensitive to both type of fluorocarbon component and gas mixing ratios due to thermally stable chamber wall conditions. Definitely, the last assumption looks to be arguable because the fraction of O_2_ may influence chamber wall conditions through the transition between polymer-rich and polymer-free states. Unfortunately, there is no reasonable theoretical approach to account for this phenomenon, and the available experimental data on recombination probabilities are for polymer-free surfaces. Therefore, one can refer only for several evidence sources that the postulation of γ = const does not make a problem, at least for given gas systems and processing conditions. For instance, Kimura et al. [12] reported the acceptable agreement between model-predicted and measured F atoms densities in CF_4_ + O_2_ plasma in the range of 0–80% O_2_. The same or even the better result was produced by our model using Kimura’s plasma diagnostics data as inputs [13]. In addition, the model of Rauf et al. [19] as well as our model [13] demonstrated the good agreement with measured densities of CF_2_ and CF radicals as functions of input power in the C_4_F_8_ plasma. Obviously, this parameter also influences the polymer deposition rate and thus affects the chamber wall condition. Finally, we obtained the evident similarity between measured and calculated F atom densities in CF_4_ + CHF_3_ + Ar and CF_4_ + C_4_F_8_ + Ar plasmas as functions of fluorocarbon gas ratios [11]. As corresponding components are featured by different polymerizing abilities, the transition between polymer-rich and polymer-free chamber wall condition surely took place.
4. The electronegativity of CF_4_, CHF_3_, C_4_F_8_, and O_2_ plasmas at p < 20 mTorr is low enough to equalize densities of electrons ($n_{e}$) and positive ions ($n_{+}$) as well as to neglect the effect of negative ions on the ion Bohm velocity [9,14,34]. In this case, the total density of positive ions may be extracted from measured $J_{+}$ simply as
  (2) $$n_{+}\approx \frac{J_{+}}{0.61e\sqrt{eT_{e}/m_{i}}}\;,$$
  where $m_{i}={\left(\sum y_{X^{+}{}_{i}}/m_{X^{+}{}_{i}}\right)}^{-1}$ is the effective ion mass while $m_{X^{+}{}_{i}}$ and $y_{X^{+}{}_{i}}$ are partial ion masses and fractions, respectively. For each type of positive ion, the last parameter may be evaluated as $y_{X^{+}}\sim k_{iz}y_{X}/\sqrt{1/m_{X^{+}}}$ [11,14], where $y_{X}$ is the fraction of corresponding neutral particle with the ionization rate coefficient of $k_{iz}=f\left(T_{e}\right)$ [12,18,19,34].

Finally, we would like to mention that used kinetic schemes and modeling approaches provide the principally correct description of plasma chemistry in given gas systems. Such an assertion is based on the general reasonability of our model-predicted data, on their qualitative similarity with those obtained by other authors, and on the agreement with experiments, as will be shown below.

**Table 1:** Basic reaction set describing the chemistry of neutral species in CF_4_ + O_2_ plasma.

Process		k	Process		k
1.	CF_x_ + e → CF_x−1_ + F + e	$f\left(T_{e}\right)$	17.	FO + O/O(^1^D) → O_2_ + F	$f\left(T_{gas}\right)$
2.	CF_x_ + e → CF_x−1_^+^ + F + 2e	$f\left(T_{e}\right)$	18.	F_2_ + O/O(^1^D) → FO + F	$f\left(T_{gas}\right)$
3.	CF_x_ + e → CF_x−2_ + 2F + e	$f\left(T_{e}\right)$	19.	CF + O_2_ → CFO + O	$f\left(T_{gas}\right)$
4.	F_2_ + e → 2F + e	$f\left(T_{e}\right)$	20.	C + O_2_ → CO + O	$f\left(T_{gas}\right)$
5.	O_2_ + e → O + O/O(^1^D) + e	$f\left(T_{e}\right)$	21.	CO + F → CFO	
6.	O + e → O(^1^D) + e	$f\left(T_{e}\right)$	22.	CF_x_ → CF_x_(s)	f(γ)
7.	CF_x_O + e → CF_x−1_O + F + e	$f\left(T_{e}\right)$		CF_x_(s) + F → CF_x+1_	
8.	FO + e → F + O + e	$f\left(T_{e}\right)$		CF_x_(s) + O → CF_x_O	
9.	CO_x_ + e → CO_x−1_ + O + e	$f\left(T_{e}\right)$	23.	F → F(s)	f(γ)
10.	F_2_ + CF_x_ → CF_x+1_ + F	$f\left(T_{gas}\right)$		F(s) + F → F_2_	
11.	CF_x_ + F → CF_x+1_	$f\left(T_{gas}\right)$		F(s) + CF_x_ → CF_x+1_	
12.	CF_x_ + O/O(^1^D) → CF_x−1_O + F	$f\left(T_{gas}\right)$		F(s) + O → FO	
13.	CF_x_ + CFO → CF_2_O + CF_x−1_	$f\left(T_{gas}\right)$	24.	O → O(s)	f(γ)
14.	2CFO → CF_2_O + CO	$f\left(T_{gas}\right)$		O(s) + CF_x_ → CF_x_O	
15.	CFO + F → CF_2_O	$f\left(T_{gas}\right)$		O(s) + O → O_2_	
16.	CF_x_O + O/O(^1^D) → F_x_ + CO_2_	$f\left(T_{gas}\right)$		O(s) + F → FO	

Notes: (a) For R1−R9, $k=AT_{e}{}^{B}exp\left(-C/T_{e}\right)$, where parameters A, B, and C are known from [12,18,28]; (b) For R10–R21, $k=A{\left(T_{gas}/298\right)}^{n}exp\left(-E_{a}/RT_{gas}\right)$, where parameters A, n and are known from [37]; (c) For R22–R24, $k\approx \left(r+l\right)\upsilon_{T}\gamma /2rl$, where $\upsilon_{T}=f\left(T_{gas}\right)$ is the thermal velocity for a gas-phase reactant, and the parameter γ is known from [12,18,29].

**Table 2:** Additive (to those specified in Table 1) reactions to describe the chemistry of neutral species in CHF_3_ + O_2_ plasma.

Process		k	Process		k
25.	CHF_x_ + e → CHF_x−1_ + F + e	$f\left(T_{e}\right)$	35.	CHF_x_ + O → CF_x_O + H	$f\left(T_{gas}\right)$
26.	CHF_x_ + e → CF_x_ + H + e	$f\left(T_{e}\right)$	36.	CHF_x_ + O → CF_x−1_O + HF	$f\left(T_{gas}\right)$
27.	CHF_x_ + e → CF_x−1_ + HF + e	$f\left(T_{e}\right)$	37.	CF_x_ → CF_x_(s)	f(γ)
28.	H_2_ + e → 2H + e	$f\left(T_{e}\right)$		CF_x_(s) + H → CHF_x_	
29.	HF+ e → H + F + e	$f\left(T_{e}\right)$	38.	F → F(s)	f(γ)
30.	F_2_ + H → HF + F	$f\left(T_{gas}\right)$		F(s) + CHF_x_ → CHF_x+1_	
31.	H_2_ + F → HF + H	$f\left(T_{gas}\right)$	39.	H → H(s)	f(γ)
32.	CF_x_ + H → CF_x−1_ + HF	$f\left(T_{gas}\right)$		H(s) + CF_x_ → CHF_x_	
33.	CHF_x_ + F → CF_x_ + HF	$f\left(T_{gas}\right)$		H(s) + H → H_2_	
34.	CHF_x_ + H → CHF_x−1_ + HF	$f\left(T_{gas}\right)$		H(s) + F → HF	

Notes: (a) For R25−R29, $k=AT_{e}{}^{B}exp\left(-C/T_{e}\right)$, where parameters A, B, and C are known from Refs. [21,29,31]; (b) For R30–R36, $k=A{\left(T_{gas}/298\right)}^{n}exp\left(-E_{a}/RT_{gas}\right)$, where parameters A, n, and $E_{a}$ are known from [37]; (c) For R37–R39, $k\approx \left(r+l\right)\upsilon_{T}\gamma /2rl$, where $\upsilon_{T}=f\left(T_{gas}\right)$ is the thermal velocity for a gas-phase reactant, and the parameter γ is known from [29,31].

**Table 3:** Additive (to those specified in Table 1) reactions to describe the chemistry of neutral species in C_4_F_8_ + O_2_ plasma.

Process		k	Process		k
40.	C_4_F_8_ + e → 2C_2_F_4_ + e	$f\left(T_{e}\right)$	45.	C_2_F_4_ + F → CF_2_ + CF_3_	$f\left(T_{gas}\right)$
41.	C_4_F_8_ + e → C_3_F_6_ + CF_2_ + e	$f\left(T_{e}\right)$	46.	C_2_F_4_ + O → CFO + CF_3_	$f\left(T_{gas}\right)$
42.	C_2_F_4_ + e → 2CF_2_ + e	$f\left(T_{e}\right)$	47.	C_2_F_4_ + O → CF_2_O + CF_2_	$f\left(T_{gas}\right)$
43.	C_2_F_4_ + e → C_2_F_3_ + F + e	$f\left(T_{e}\right)$	48.	F → F(s)	f(γ)
44.	C_2_F_3_ + e → CF_2_ + CF + e	$f\left(T_{e}\right)$		F(s) + C_2_F_3_ → C_2_F_4_	

Notes: (a) For R40−R44, $k=AT_{e}{}^{B}exp\left(-C/T_{e}\right)$, where parameters A, B, and C are known from [18,19,30]; (b) For R45–R47, $k=A{\left(T_{gas}/298\right)}^{n}exp\left(-E_{a}/RT_{gas}\right)$, where parameters A, n, and $E_{a}$ are known from [37]; (c) For R48, $k\approx \left(r+l\right)\upsilon_{T}\gamma /2rl$, where $\upsilon_{T}=f\left(T_{gas}\right)$ is the thermal velocity for a gas-phase reactant, and the parameter γ is known from [18,19,20].

### 2.3. Approaches for the Analysis of Etching/Polymerization Kinetics

To analyze the Si etching kinetics as well as to determine etching mechanism, we used the phenomenological approach discussed in our previous works [10,11,13,14,20,23]. The basic concept was developed in [4,38,39,40,41] and may be summarized as follows:

1. When the ion bombardment energy exceeds the sputtering threshold, the total etching rate R may be represented as a combination of two summands, $R_{phys}+R_{chem}$ [40], where $R_{phys}$ and $R_{chem}$ are rates of physical sputtering and heterogeneous chemical reaction supplied by neutral etchant species. Since the latter sometimes exhibits the nonzero energy threshold and/or leads to the formation of low volatile reaction products, the dependence of $R_{chem}$ on processing conditions may be sensitive to energy fluxes coming with nonreactive species, in particular with positive ions. Such processes are known as the ion-assisted chemical reaction.
2. The rate of physical sputtering $R_{phys}=Y_{S}\Gamma_{+}$ [40,41], where $Y_{S}$ ~ $\sqrt{\;M_{i}\varepsilon_{i}}$ [9,10,11] is the sputter yield, $\;M_{i}=m_{i}N_{A}$ is the effective (ion-type-averaged) ion molar mass, $\varepsilon_{i}=\left|-U_{f}-U_{dc}\right|$ is the ion bombardment energy, $-U_{f}\approx 0.5T_{e}\ln\left(m_{i}/2\pi m_{e}\right)$ is the floating potential, and $\Gamma_{+}\approx J_{+}/e$ is the flux of positive ions. Accordingly, the parameter $\sqrt{M_{i}\varepsilon_{i}}\Gamma_{+}$ adequately traces the behavior of $R_{phys}$ with variation of processing conditions [9,11,12]. The similar rule can also be applied to other ion-driven effects on the etched surface, such as the removal of the fluorocarbon polymer film, the destruction of chemical bonds between surface atoms, and the ion-stimulated desorption of low volatile reaction products.
3. The rate of heterogeneous chemical reaction $R_{chem}=\gamma_{R}\Gamma_{F}$ [22,23,32,40], where $\Gamma_{F}\approx n_{F}\upsilon_{T}/4$ is the fluorine atom flux, $\gamma_{R}\approx s_{0}\left(1-\theta \right)$ is the effective reaction probability [9,10,11], θ is the fraction of adsorption sites occupied by chemically inert species, (1−θ) is the fraction of vacant adsorption sites, and $s_{0}$ is the sticking coefficient of etchant species to the vacant adsorption site. That is why the parameter $\gamma_{R}$ is not only the exponential function of surface temperature $T_{S}$ (as it typically takes place for the spontaneous reaction mechanism) but also depends on many plasma-related factors that retard or accelerate the chemical reaction through the change in (1−θ). For instance, in strongly polymerizing plasmas, $\gamma_{R}$ decreases with increasing polymer film thickness, as the latter becomes to be enough to provide ${\Gamma_{F}}^{’}/\Gamma_{F}$ << 1, where ${\Gamma_{F}}^{’}$ is the flux of F atoms on the polymer film/etched surface interface. As such, the correlation of $\gamma_{R}$ with fluxes of plasma active species at $T_{S}$ = const provides useful information on the mechanism of chemical etching pathway.
4. The formation of the fluorocarbon polymer film is provided by nonsaturated CH_x_F_y_ (x + y < 3) radicals while the polymerization ability increases under the fluorine-poor conditions [6,7,8]. Accordingly, the $\Gamma_{pol}/\Gamma_{F}$ ratio, where $\Gamma_{pol}$ is the total flux of polymerizing radicals, traces the polymer deposition rate while parameters $\Gamma_{pol}/\sqrt{M_{i}\varepsilon_{i}}\Gamma_{+}\Gamma_{F}$ and $\Gamma_{pol}/\Gamma_{O}\Gamma_{F}$ reflect relative changes in the polymer film thickness due to physical (destruction by ion bombardment) and chemical (etching by O atoms) mechanisms [9,11,14].

## 3. Results and Discussion

**Figure 1:**
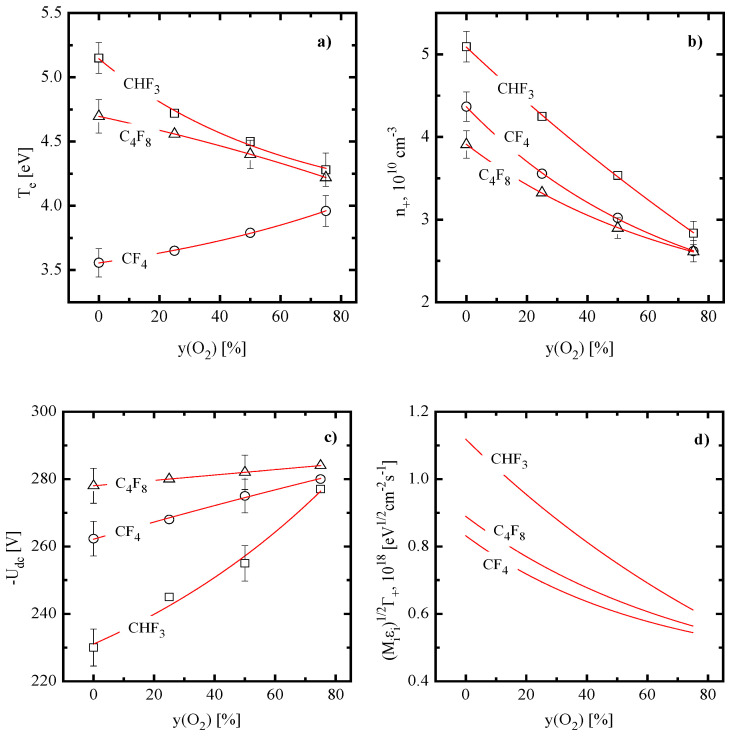
Electrons- and ions-related plasma parameters vs. fraction of O_2_ in CF_4_ + O_2_, CHF_3_ + O_2_, and C_4_F_8_ + O_2_ plasmas: (**a**) electron temperature; (**b**) plasma density; (**c**) negative dc bias voltage; and (**d**) parameter $\sqrt{M_{i}\varepsilon_{i}}\Gamma_{+}$ characterizing the ion bombardment intensity. Processing conditions are: gas pressure of 6 mTorr, input power of 700 W and bias power of 200 W. Sections (**a**–**c**) represent experimental data while solid lines are their fittings to guide the eye only. Section (**d**) represents model-predicted data.

**Figure 2:**
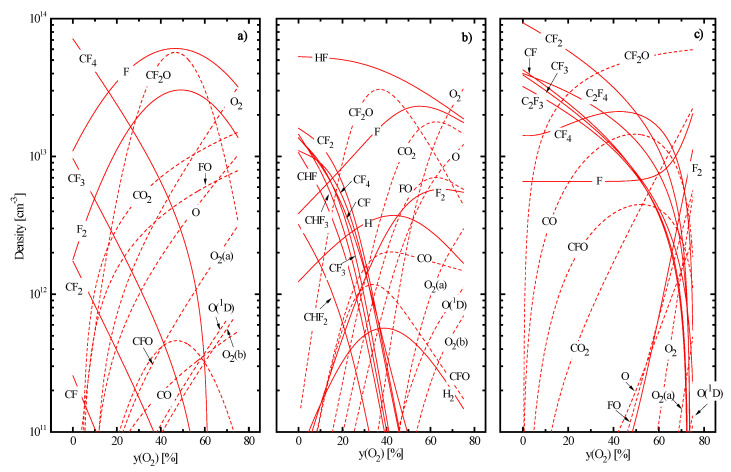
Model-predicted densities of neutral species vs. fraction of O_2_ in CF_4_ + O_2_ (**a**), CHF_3_ + O_2_ (**b**) and C_4_F_8_ + O_2_ (**c**) plasmas. Curves marked as O_2_ (**a**) and O_2_ (**b**) correspond to metastable states of O_2_(a^1^Δ) and O_2_(b^1^Σ), respectively. Dashed lines are to highlight oxygen-containing reaction products. Processing conditions are the same as those specified under Figure 1.

The basic properties of nonoxygenated CF_4−_, CHF_3−_ and C_4_F_8−_ plasmas have been studied, analyzed, and compared in our previous works [9,10,11,20]. As such, the discussion below will cover only the issues that seem to be important for understanding plasma chemistry and etching kinetics in the presence of oxygen. In addition, since it was impossible to perform the quantitative analysis for both electron energy loss channels and ionization/recombination balance (see our arguments in Section 2.2), the effect of O_2_ on both electron temperature and plasma density may be discussed only in a suggestive scale, with accounting for general regularities of plasma chemistry and model-predicted densities of neutral species. As such, plasma diagnostics data shown in Figure 1 may briefly be commented as follows:

- Electron temperature (Figure 1a) exhibits a weak growth in the CF_4_ + O_2_ plasma (3.6–4.0 eV for 0–75% O_2_) but decreases gradually in both CHF_3_ + O_2_ (5.2–4.3 eV for 0–75% O_2_) and C_4_F_8_ + O_2_ (4.7–4.2 eV for 0–75% O_2_) plasmas. Perhaps, the first phenomenon is caused by increasing fraction of atomic species, as shown in Figure 2a. As collisions with molecules surely provide higher electron energy losses for both excitation (due to the low-threshold vibrational and electronic states) and ionization (due to generally higher ionization cross-sections for bigger-sized particles), a decrease in the overall electron energy loss takes place. Accordingly, the opposite situation in the CHF_3_ + O_2_ plasma reflects increasing electron energy losses, since decreasing tendency for originally dominating HF molecules meets the growth of multiatomic reaction products, such as FO, CF_x_O, and CO_x_ (Figure 2b). For instance, CO_2_ has three vibrational modes [42], and corresponding cross-sections are featured by higher absolute values and wider maximum compared with those for HF [42,43]. Probably, the similar mechanism also does work in the C_4_F_8_ + O_2_ plasma, where an increase in $y_{O_{2}}$ changes the dominant gas-phase component from CF_2_ radicals to CF_4_, CO, CO_2_, and CF_2_O (Figure 2c).
- Plasma density (Figure 1b) exhibits decreasing tendencies vs. $y_{O_{2}}$ in all three gas systems. In the case of CF_4_ + O_2_ plasma, the evident reason is the 10-times lower rate coefficients for the ionization of F (~5.8 × 10^−11^ cm^3^/s at $T_{e}$ = 3 eV) and F_2_ (~1.5 × 10^−11^ cm^3^/s at $T_{e}$ = 3 eV) compared with CF_x_ (~1.5 × 10^−10^ cm^3^/s for x = 4 and ~5.0 × 10^−10^ cm^3^/s for x = 3 at $T_{e}$ = 4 eV). Therefore, one can easily imagine that an increases in $y_{O_{2}}$ suppresses the total ionization frequency (and thus production rates for electrons and positive ions) despite weakly increasing $T_{e}$. Similar situations for CHF_3_ + O_2_ and C_4_F_8_ + O_2_ plasmas probably result from decreasing ionization rate coefficients for all neutral species. The indirect proof is the deeper fall of $n_{+}$ in the CHF_3_ + O_2_ plasma, where the stronger decrease in $T_{e}$ takes place. An additional reason may relate to increasing densities of more electronegative oxygen-containing species that accelerates losses of positive ions and electrons through ion–ion recombination and dissociative attachment, respectively.
- Negative dc bias voltage (Figure 1c) demonstrates the monotonic growth vs. $y_{O_{2}}$ in all three gas systems. This is because the decreasing ion flux (as it directly follows from the change in $n_{+}$) weakens the compensation for an excess negative charge under the condition of $W_{dc}$ = const. At the same time, weak increase in ion bombardment energies ($\varepsilon_{i}$= 285–303 eV for CF_4_ + O_2_, 262–302 eV for CHF_3_ + O_2_, and 306–309 eV for C_4_F_8_ + O_2_ at 0–75% O_2_) is overcompensated by opposite tendencies of both $\Gamma_{+}$ and effective ion masses. As a result, the parameter $\sqrt{M_{i}\varepsilon_{i}}\Gamma_{+}$ always demonstrates the monotonic decrease toward O_2_-rich plasmas (Figure 1d). As such, the common feature is that the addition of O_2_ reduces the ion bombardment intensity.

Based on the above data on electrons-related plasma parameters, we performed the analysis of plasma chemistry with the focus on the fluorine atoms kinetics.

**Figure 3:**
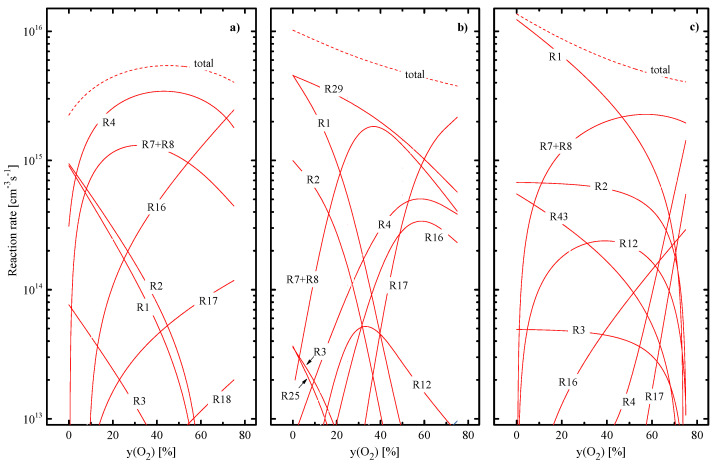
Model-predicted F atom formation rates vs. fraction of O_2_ in CF_4_ + O_2_ (**a**), CHF_3_ + O_2_ (**b**), and C_4_F_8_ + O_2_ (**c**). Labels on curves correspond to reaction numbers in Table 1, Table 2 and Table 3. Processing conditions are the same as those specified under Figure 1.

In pure CF_4_ plasma, dominant gas-phase components are original CF_4_ molecules, CF_3_ radicals, and fluorine atoms with the condition of $n_{CF_{4}}$ > $n_{CF_{3}}$ ≈ $n_{F}$ [9,10,11] (Figure 2a). Accordingly, reactions R1 for x = 3, 4 and R2 for x = 4 compose ~ 80% of the total F atom formation rate (Figure 3a). The loss of F atoms is mainly provided by heterogeneous recombination pathways, such as F + F → F_2_ and F + CF_x_ → CF_x+1_ inside R22 and R23. The addition of O_2_ rapidly lowers densities of CF_x_ radicals due to their conversion into CF_2_O, CFO, CO and CO_2_ species (Figure 2a) in R12 ($k_{12}$ ~ 6.1 × 10^−11^ cm^3^/s for x = 1 and ~3.2 × 10^−11^ cm^3^/s for x = 2, 3). The domination of CF_2_O over oxygen-containing reaction products as well as the drastic growth of their density at $y_{O_{2}}$ < 40% is supported by gas-phase processes R13 ($k_{13}$ ~ 1.1 × 10^−11^ cm^3^/s for x = 2 and ~ 7.0 × 10^−13^ cm^3^/s for x = 1), R14 ($k_{14}$ ~ 1.0 × 10^−11^ cm^3^/s) and R15 ($k_{15}$ ~ 8.0 × 10^−11^ cm^3^/s). The condition $y_{O_{2}}$ > 40% causes the more than ten-time decrease in $n_{CF_{x}}$ as well as reduces rates of R12 and R13 due to the fluorocarbon-deficient reaction regime. Accordingly, this produces the maximum on the $n_{CF_{2}O}=f\left(y_{O_{2}}\right)$ curve, as shown in Figure 2a. As for the kinetics of F atoms, there are two principal effects influencing their formation rate. First, an increase in $y_{O_{2}}$ accelerates the formation of F_2_ molecules through R16 ($k_{16}$ ~ 2.0×10^−11^ cm^3^/s for x = 2) and R23 that sufficiently influences the rate of R4 (Figure 3a). As a result, the latter overlaps the total effect from R1 and R2 at $y_{O_{2}}\;$> 10–15% O_2_ as well as exhibits the maximum at ~40% O_2_ following the change in $n_{F_{2}}$. Second, the appearance of oxygen adds new F atom formation pathways, such as R7, R8, R12, and R16–R18. From Figure 3a, it can be understood that the superposition of R4, R7, and R8 produces the maximum on the total F atom formation rate (and thus on the F atom density, as shown in Figure 2a) while noticeable contributions of R16 and R17 appear only at $y_{O_{2}}$ > 60%. The faster growth of $n_{F}$ at 0–40% O_2_ is due to the simultaneously decreasing F atom losses in F + CF_x_ → CF_x+1_ pathways inside R22 and R23.

In pure CHF_3_ plasma, dominant gas-phase components are HF molecules and CF_x_ (x = 1, 2) radicals (Figure 2b). The phenomenon of HF is due to the effective formation of these species in gas-phase reactions R32 ($k_{32}$~1.2 × 10^−11^ cm^3^/s for x = 1, ~2.2 × 10^−11^ cm^3^/s for x = 2 and ~7.9 × 10^−11^ cm^3^/s for x = 3), R33 ($k_{33}$~3.3 × 10^−11^ cm^3^/s for x = 1 and 2 while ~1.6 × 10^−13^ for x = 3), and R34 ($k_{34}$ ~3.1 × 10^−10^ cm^3^/s for x = 1 and 2). Accordingly, the decomposition of HF in R29 represents the essential (~45%) part of the total F atom formation rate while almost the same contribution comes from the couple of R1 and R2 (Figure 3b). Another important feature is that the loss of F atoms through R33 dominates over their heterogeneous recombination in R22, R23, R38, and R39. That is why the CHF_3_ plasma exhibits the higher F atom formation rate compared with CF_4_ (due to the condition of $k_{29}$ > $k_{1}$ + $k_{2}$ for x = 4) but is characterized by the lower F atom density. As in the previous case, the addition of O_2_ suppresses densities of fluorocarbon radicals as well as increases the density of F atoms (Figure 2b). The first effect is due to the oxidation of both CF_x_ and CHF_x_ radicals into CF_x_O species in R12, R35 ($k_{35}$ ~ 1.1 × 10^−11^ cm^3^/s for x = 2), and R36 ($k_{36}$ ~3.5 × 10^−11^ cm^3^/s for x = 1). In particular, the above three processes together with R21, R23, and R24 provide increasing densities of FO and CFO molecules, and the consumption of CFO in R13–R15 produces CF_2_O. That is why the latter becomes the essential gas-phase component in the range of 20–60% O_2_. From Figure 3b, it can be understood also that the total add-on from R4, R7, and R8 to the total F atom formation rate does not overcome the level of R29 but only compensates for decreasing rates of R1 and R2. The contribution from atom–molecular reactions R12, R16, and R17 overtakes R29 at $y_{O_{2}}$ > 50%, but their cumulative effect does not reach the level of R29 in pure CHF_3_ plasma. As such, one can obtain the decreasing tendency for the total F atom formation rate (Figure 3b) that contradicts with increasing F atom density (Figure 2b). Such a phenomenon is due to the more rapid decrease in the F atom loss frequency in R33 due to decreasing densities of CHF_x_ species. The similar mechanism was found to explain the very slow decrease in the F atom density with increasing Ar fraction in the CHF_3_ + Ar plasma [9,10]. Another principal difference of CHF_3_ + O_2_ gas system compared with CF_4_ + O_2_ is that an increase in $y_{O_{2}}$ causes the slower growth of R5 due to a decrease in both $T_{e}$ and $n_{e}$. This limits reaction rates with a participation of O and O(^1^D) species, including those resulting in the production of F atoms.

In pure C_4_F_8_ plasma, the gas phase is mostly composed by nonsaturated fluorocarbon radicals CF_x_ (x = 1, 2, 3) and C_2_F_x_ (x = 3, 4) (Figure 2c). These particles appear from original C_4_F_8_ molecules in R40 and R41 as well as result from the further decomposition of corresponding reaction products through R1 for x = 2 and R42–R44. Accordingly, the main source of F atoms is R1 for x = 1–3 (Figure 3c) while their decay, in addition to heterogeneous processes R22, R23, and R48, is noticeably contributed by R45 ($k_{45}$ ~ 4.0 × 10^−11^ cm^3^/s). Due to the last process, the C_4_F_8_ plasma exhibits the relatively low $n_{F}$ value compared with that for CF_4_ (Figure 2c) thought is characterized by higher F atom formation rate (Figure 3c). The addition of O_2_ also initiates the decomposition of CF_x_ radicals through R12 but results in much weaker decrease in their densities compared with CF_4_ + O_2_ plasma (Figure 2c). The reason is the effective loss of O_2_ molecules in R19 ($k_{19}$ ~ 3.2 × 10^−11^ cm^3^/s) and R20 ($k_{20}$ ~ 1.5 × 10^−11^ cm^3^/s) that provides their conversion into CO and CO_2_ species. As both CO and CO_2_ have lower dissociation rate coefficients compared with O_2_ itself (for example, ~5.2 × 10^−10^ cm^3^/s for R9 with x = 1 vs. ~3.1 × 10^−9^ cm^3^/s for R5 with a formation of O(^1^D)), such situation results in sufficiently lower O and O(^1^D) densities compared with CF_4_ + O_2_ plasma at identical $y_{O_{2}}$ values. The lack of active oxygen reduces the significance of R12, R16 and R17 in respect to the production of F atoms in the O_2_-rich plasmas as well as leads to a decrease in the total F atom formation rate toward higher $y_{O_{2}}$ values, as shown in Figure 3c. At the same time, one can also obtain the rapid decrease in the F atom loss frequency in R45 due to the same change in the density of corresponding source species, C_2_F_4_. The slower fall of $n_{C_{2}F_{4}}$ at $y_{O_{2}}$ < 50% (by ~ 4 times for 0–50% O_2_) just compensates for a decrease in the total F atom formation rate and thus results in $n_{F}$ ≈ const. Accordingly, the faster decrease in $n_{C_{2}F_{4}}$ at $y_{O_{2}}$ > 50% (by more than 1000 times for 50–75% O_2_) causes the growth of F atom density. It should be mentioned that our kinetic data for CF_4_ + O_2_ and C_4_F_8_ + O_2_ plasmas are in principal agreement with experimental results of Hayashi et al. [44]. In particular, they also obtained that the last gas system exhibits much higher density of CF_2_ radicals as well as is featured by the lower density of O atoms. Moreover, their conclusion was that an essential O atom loss pathway in the C_4_F_8_ + O_2_ plasma is namely their interaction with CF_2_. Accordingly, our model also shows that R12 for x = 2 together with O + CF_2_ → CF_2_O channels inside R22 and R44 provide the dominant part of the total O atom loss rate at $y_{O_{2}}$ > 20%. Therefore, data of Figure 3 adequately reflect the real situation. 

**Figure 4:**
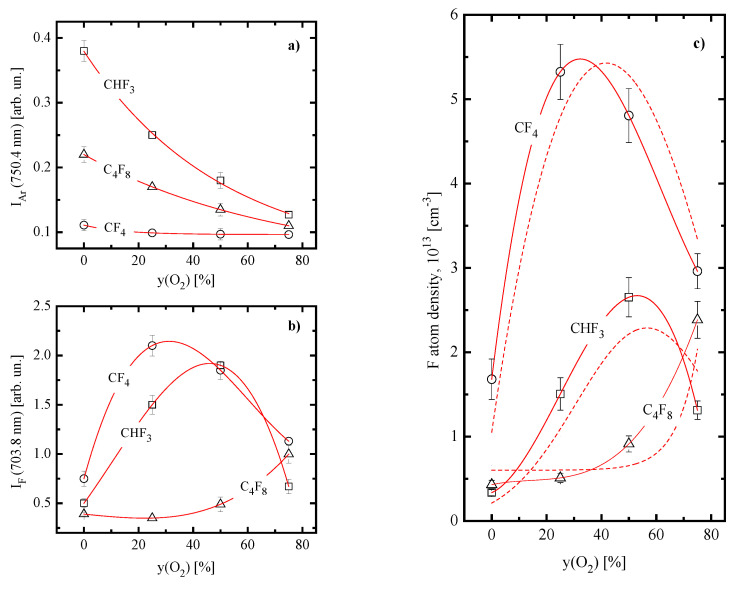
Emission intensities (Ar 750.4 nm (**a**) and F 703.8 nm (**b**)) as well as F atom densities obtained using the actinometry procedure (**c**) vs. fraction of O_2_ in CF_4_ + O_2_, CHF_3_ + O_2_ and C_4_F_8_ + O_2_ plasmas. Solid lines in sectons (**a**–**c**) are fittings of experimental data to guide the eye only. Dashed lines in Figure (**c**) repeat model-predicted F atom densities from Figure 2. Processing conditions are the same with those specified under Figure 1.

Figure 4 illustrates the results of plasma diagnostics by OES as well as compares model-predicted and measured F atom densities. From Figure 4a, it can be seen that the intensity of Ar 750.4 nm line depends on both type of fluorocarbon gas and $y_{O_{2}}$ even at = const. This fact reasonably reflects the nonconstancy of excitation functions $k_{ex,Ar}n_{e}$, where $k_{ex,Ar}=f\left(T_{e}\right)$ is the excitation rate coefficient. When returning back to Figure 1, one can conclude that the behavior of $I_{Ar}$ confirms the general reasonability of plasma parameters obtained after the diagnostics by Langmuir probes. Really, the very weak change of $I_{Ar}$ in the CF_4_ + O_2_ plasma agrees with the nearly constant $k_{ex,Ar}n_{e}$ (2.2–2.0 s^−1^ for 0–75% Ar) produced by opposite changes in electron temperature and electron density. Accordingly, the highest $I_{Ar}$ as well as the sharper slope for $I_{Ar}=f\left(y_{O_{2}}\right)$ curve in the CHF_3_ + O_2_ plasma are due to the stronger change in $k_{ex,Ar}n_{e}$ (8.1–2.2 s^−1^ for 0–75% Ar), as follows from features of electron temperature and electron density. It is clear also that emission intensities for F 703.8 nm line (Figure 4b) represent the overall effect from two factors, such as excitation function and F atom density. From Figure 4c, it can be understood that that model-predicted densities of F atoms in all three gas mixtures are in the satisfactory agreement with those obtained by the actinometry. The fact that measured and model-yielded $n_{F}=f\left(y_{O_{2}}\right)$ curves for each gas mixture are shifted one in respect to each other by more than the experimental error does not contradict the above conclusion. The reason is that both model and experiment are based on many approaches as well as involve many kinetic data (in particular, process cross-sections) which also are characterized by their own errors. Therefore, the principally important thing is that that the actinometry confirms model-predicted behaviors of F atoms densities as well as does not result in abnormal differences in corresponding absolute values. The latter means that constant recombination probabilities for atoms and radicals in the plasma modeling procedure are really not a problem for the model adequacy issue. Nevertheless, though this conclusion is valid for all three gas mixtures, the physical reasons may be different. In the CF_4_ + O_2_ plasma, one can suggest no sufficient differences in chamber wall conditions for O_2−_pure and O_2_-rich gas mixtures due to the low polymerizing ability for CF_4_ itself. As such, recombination probabilities may really be nearly constant. In both CHF_3_ + O_2_ and C_4_F_8_ + O_2_ plasmas, the loss of atoms and radicals is sufficiently (sometimes—dominantly, as was mentioned above) contributed by gas-phase processes. As a result, steady-state densities of corresponding species appear to be low sensitive to changes in heterogeneous recombination kinetics, if those really take place. For instance, the twofold increase in F atom recombination probability causes the less than 10% response in model-predicted species densities in both O_2−_pure and O_2_-rich C_4_F_8_ + O_2_ plasmas. Finally, we would like to underline that fluorine atoms are closely matched with other species through both gas-phase and heterogeneous reactions. Therefore, data of Figure 4c reveal that our plasma diagnostic and model-based analysis of plasmas chemistry provides the correct understanding of main kinetic effects influencing the steady-state plasma composition.

**Figure 5:**
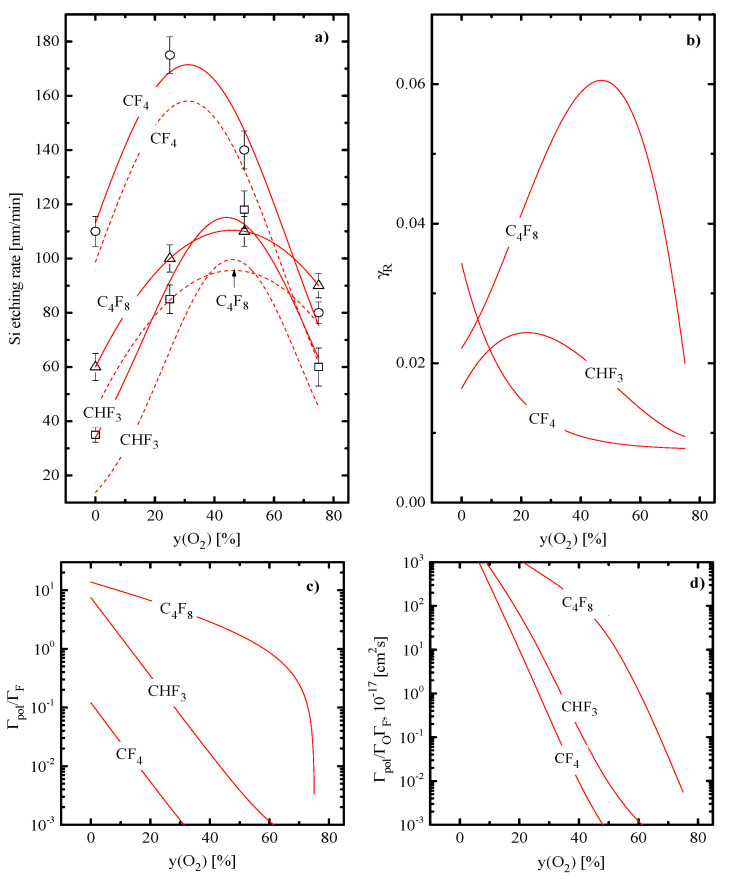
Silicon etching kinetics and flux-to-flux ratios characterizing polymerization effects vs. fraction of O_2_ in CF_4_ + O_2_, CHF_3_ + O_2_ and C_4_F_8_ + O_2_ plasmas: (**a**) measured Si etching rates (solid lines + symbols) and model-predicted $R_{chem}$ (dashed lines); (**b**) effective reaction probabilities; (**c**) $\Gamma_{pol}/\Gamma_{F}$ ratios characterizing polymer deposition rate; (**d**) $\Gamma_{pol}/\Gamma_{O}\Gamma_{F}$ ratios characterizing the change in polymer film thickness due to its chemical etching by oxygen atoms. Solid lines in section (**a**) are fittings of experimental data to guide the eye only. Processing conditions are the same as those specified under Figure 1.

From Figure 5a, it can be seen that the Si etching rates in CF_4_ + O_2_, CHF_3_ + O_2_ and C_4_F_8_ + O_2_ plasmas are characterized by different absolute values but exhibit similar nonmonotonic changes with increasing vs. $y_{O_{2}}$. The evaluation of $R_{phys}=Y_{S}\Gamma_{+}$ using experimental data on Si sputtering yields ($Y_{S}$ ≈ 0.18–0.29 atom/ion at ion energies of 200–300 eV [41,45]) indicates that the condition $R_{phys}$ << R, where R is the measured Si etching rate, always takes place. The latter means that (a) the dominant etching mechanism in all three cases is the heterogeneous chemical reaction R49: Si(s.) + xF → SiF_x_(s.) → SiF_x_ (where (s.) relate to the surface-bonded state), characterized by the rate of $R_{chem}=R-R_{phys}$; (b) the nonmonotonic $R=f\left(y_{O_{2}}\right)$ curves are completely related to identical behaviors of $R_{chem}$; and (c) the shape of $R_{chem}=f\left(y_{O_{2}}\right)$ curves in C_4_F_8_ + O_2_ plasma contradicts with the monotonic change in the F atom flux that directly points out on the nonconstant effective reaction probability $\gamma_{R}\approx R_{chem}/\Gamma_{F}$. In reality, it was found that all three reaction probabilities are sensitive to the O_2_ fraction in a feed gas even at the nearly constant surface temperature (Figure 5b).

In our opinion, the corresponding phenomena may be explained as follows:

- In weakly-polymerizing CF_4_ + O_2_ plasma (as it combines lowest polymer deposition rate and the highest polymer etching rate by oxygen atoms, as follows from Figure 5c,d), the behavior of $\gamma_{R}$ contradicts with decreasing polymer film thickness but demonstrates the similar change with the parameter $\sqrt{M_{i}\varepsilon_{i}}\Gamma_{+}$. At the same time, such correlation seems to be the formal thing, since the spontaneous mechanism of R49 must be lowly sensitive to the ion bombardment intensity [4,45]. Moreover, an increase in the ion bombardment intensity may even lower the Si + F reaction probability due to the ion-stimulated desorption of etchant species [46,47,48]. Therefore, when assuming the rather thin or the noncontinuous fluorocarbon polymer film which does not influence the etching kinetics, the most realistic reason is the passivation of etched surface by oxygen atoms. The latter may either work through the oxidation of silicon as R50: Si(s.) + O → SiO(s.) or appear due to the transformation of reaction products into lower volatile compounds in R51: SiF_x_(s.) + yO → SiF_x_O_y_(s.). In particular, the first mechanism suppresses the silicon etching rate in the O_2_-rich SF_6_ + O_2_ plasmas [49,50] while the second phenomenon produces the side-wall passivation layer in cryogenic etching processes [51,52]. Therefore, even if SiF_x_O_y_ still exhibits the spontaneous desorption at nearly room temperatures [52], the corresponding resorption yield is expected to be lower compared with that for the nonoxidized SiF_x_. Anyway, it is clear that an increase in $y_{O_{2}}$ accelerates R50 and R51 but reduces the efficiency of ion-assisted reverse processes, such as R52: SiO(s.) → Si(s.) + O and R53: SiF_x_O_y_(s.) → SiF_x_O_y_. That is why an increase in $y_{O_{2}}$ suppresses $\gamma_{R}$ through decreasing fraction of free adsorption sites for F atoms. It is important to mention that our model-predicted $\gamma_{R}$ for pure CF_4_ plasma (~0.034, see Figure 5b) surely fits the range obtained in experiments with the independent sources of fluorine atoms in the absence of ion bombardment [53]. In fact, this confirms the above conclusions on the polymer-film-independent etching regime as well as on the domination of the chemical etching pathway in a form of the mainly spontaneous R49.
- In moderately polymerizing CHF_3_ + O_2_ plasma (as it is characterized by intermediate values for both polymer deposition rate and the polymer etching rate by oxygen atoms), the condition of $y_{O_{2}}$~25–30% probably corresponds to the transition from the polymer-film-dependent to the polymer-film-independent etching regime. In particular, in the CHF_3−_ rich plasma, the polymer film may be thick enough to limit the rate of R49 through the transport of F atoms to the etched surface. Accordingly, an increase in $\gamma_{R}$ up to 25–30% O_2_ reflects the opposite change in the polymer film thickness due to decreasing polymer deposition rate (Figure 5c) and increasing polymer removal rate (Figure 5d). From Figure 5c, it can be understood that the addition of 30% O_2_ lowers the polymer deposition rate down to the value obtained in pure CF_4_ plasma. As such, the similarly thin polymer film on the Si surface does remain, and the further decrease in the amount of residual polymer does not influence $\gamma_{R}$. Simultaneously, the increasing flux of oxygen atoms stimulates R50 and R51 and thus lowers the effective reaction probability for F atoms through the decreasing fraction of free adsorption sites. Therefore, the nonmonotonic shape of $\gamma_{R}=f\left(y_{O_{2}}\right)$ is due to the change in the process limiting stage.
- In strongly polymerizing C_4_F_8_ + O_2_ plasma (as it combines the highest polymer deposition rate and the lowest polymer etching rate by oxygen atoms), the thick polymer film expectedly exists even at higher $y_{O_{2}}$ values. Accordingly, the bend point on the $\gamma_{R}=f\left(y_{O_{2}}\right)$ curve at ~ 50% O_2_ also corresponds to the transition between two etching regimes where the effective probability of R49 is controlled by different factors. Similarly, to the previous case, these are either the transport of F atoms through the thick polymer film or the passivation of the etched surface by the cumulative action of R50 and R51.

Finally, we would like to note that the above data on $\gamma_{R}$ are in good agreement with those obtained in our previous work [14] for CF_4_ + Ar + O_2_, CHF_3_ + Ar + O_2_ and C_4_F_8_ + Ar + O_2_ gas mixtures. In particular, when substituting of Ar by O_2_ at constant 50% fraction of fluorocarbon component, we also detected the continuously decreasing $\gamma_{R}$ in the CF_4−_ based plasma, obtained the non-monotonic $\gamma_{R}=f\left(y_{O_{2}}\right)$ curve in the CHF_3−_ based plasma as well as found the continuously increasing $\gamma_{R}$ in the C_4_F_8−_ based plasma. Obviously, the latter does not contradict with recent data since the end point in [14] was just 50% C_4_F_8_ + 50% O_2_. Therefore, one can surely suggest that the chemical etching pathway of silicon in fluorocarbon/oxygen gas systems is always influenced by two fundamental factors, such as F atom transport through the polymer film (if the latter has an essential thickness determined by a specific combination of polymer deposition and etching rates) and oxygen-related passivation effects on either polymer-free or covered by the thin polymer film surface. The competition between these factors as well as the domination of one of these under the given set of processing conditions may be adjusted by both types of fluorocarbon component and the content of O_2_ in a feed gas. From the above data, it can also be seen that the O_2_ mixing ratio is principally important variable parameter because it changes gas-phase reaction mechanisms, adds new essential components, and influences the Si etching kinetics not only through the density of etchant species. Though all these formally relate only for the fixed combination of p, W, and $W_{dc}$ used in the given study, corresponding regularities seem to be fundamental things that are valid for wider ranges of pressures and input powers. At least, the nonsystematic test experiments indicated that all experimental and model-predicted tendencies look similar at p = 4–12 mTorr and W = 400–900 W. The latter means no changes in basic reaction mechanisms and dominant processes determining both electron energy balance and steady-state plasma composition.

## 4. Conclusions

In this work, we performed the comparative study of electrophysical plasma parameters, densities of active species, and silicon etching kinetics in CF_4_ + O_2_, CHF_3_ + O_2_, and C_4_F_8_ + O_2_ gas mixtures with variable initial compositions. The combination of plasma diagnostics by Langmuir probes, optical emission spectroscopy, and plasma modeling confirmed known features of individual fluorocarbon gases as well as allowed one to figure out key chemical processes determining plasma parameters in the presence of oxygen. It was shown that, under the investigated set of processing conditions, an increase in O_2_ content in a feed gas (a) always disturbs electrons- and ions-related plasma parameters (identically for plasma density and non-identically for electron temperature, as follows from expected changes in total ionization rates and electron energy losses); (b) results in faster, compared with the dilution effect, decrease in densities of fluorocarbon radicals due to their oxidation into CF_x_O, FO, and CO_x_ compounds; and (c) sufficiently influences the kinetics of fluorine atoms. In particular, the nonmonotonic (with a maximum at ~40–50% O_2_) change in the F atom density in the CF_4_ + O_2_ plasma repeats behavior of their formation rate after the contribution of processes involving CF_x_O и FO species. At the same time, corresponding effects for both CHF_3_ + O_2_ and C_4_F_8_ + O_2_ plasmas contradict with changes in F atom formation rates but results from decreasing decay frequency in gas-phase atom-molecular processes. These are CHF_x_ + F → CF_x_ + HF and C_2_F_4_ + F → CF_2_ + CF_3_, respectively. From etching experiments, it was found that (a) the dominant etching mechanism for Si in all three gas systems is the ion-assisted chemical reaction and (b) the nearly constant surface temperature does not mean the constant Si + F reaction probability. In fact, the latter depends on the polymerizing ability for the given fluorocarbon gas as well as appears to be sensitive to the O atom flux through both change in the thickness of fluorocarbon polymer film and oxidation of silicon surface. The domination of last mechanism lowers the Si + F reaction probability in O_2_-rich CF_4_ + O_2_ plasmas while their competition produces nonmonotonic behaviors of reaction probabilities in CHF_3_ + O_2_ (with the maximum at ~25–30% O_2_) and C_4_F_8_ + O_2_ (with the maximum at ~50% O_2_) plasmas.

## Data Availability

Not applicable.
